# Dynamics of Two Distinct Exciton Populations in Methyl-Functionalized Germanane

**DOI:** 10.1021/acs.nanolett.1c04357

**Published:** 2022-01-20

**Authors:** Eugenio Cinquanta, Samim Sardar, Warren L. B. Huey, Caterina Vozzi, Joshua E. Goldberger, Cosimo D’Andrea, Christoph Gadermaier

**Affiliations:** † Istituto di Fotonica e Nanotecnologie, Consiglio Nazionale delle Ricerche, Piazza Leonardo da Vinci 32, Milano 20133, Italy; ‡ Center for Nano Science and Technology @PoliMi, Istituto Italiano di Tecnologia, Via Giovanni Pascoli 70, Milano 20133, Italy; § Department of Chemistry and Biochemistry, The Ohio State University, Columbus, Ohio 43210, United States; ∥ Dipartimento di Fisica, Politecnico di Milano, Piazza Leonardo da Vinci 32, Milano 20133, Italy

**Keywords:** 2D materials, germanane, excitons, time-resolved photoluminescence, intercalation, ultrafast spectroscopy

## Abstract

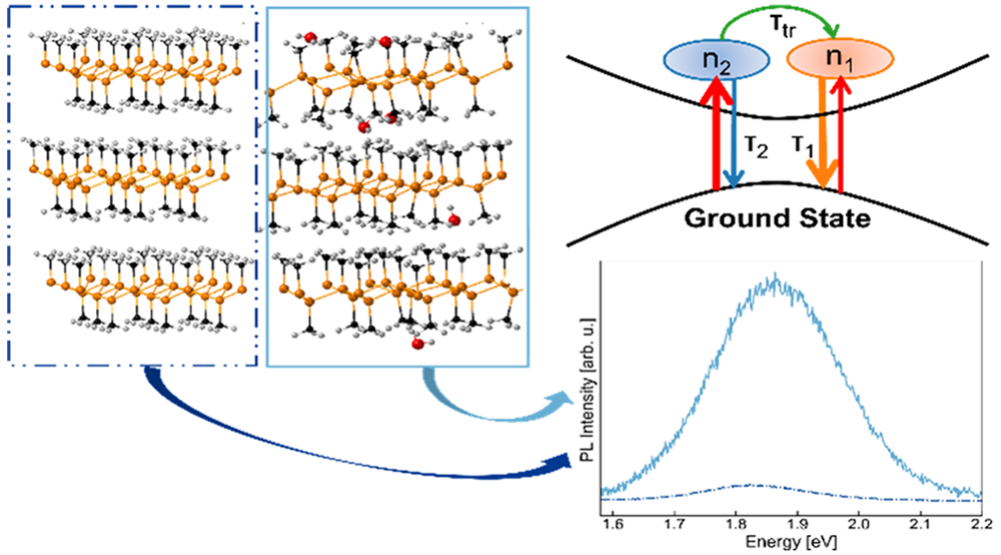

Methyl-substituted germanane is an emerging material that has been proposed for novel applications in optoelectronics, photoelectrocatalysis, and biosensors. It is a two-dimensional semiconductor with a strong above-gap fluorescence associated with water intercalation. Here, we use time-resolved photoluminescence spectroscopy to understand the mechanism causing this fluorescence. We show that it originates from two distinct exciton populations. Both populations recombine exponentially, accompanied by the thermally activated transfer of exciton population from the shorter- to the longer-lived type. The two exciton populations involve different electronic levels and couple to different phonons. The longer-lived type of exciton migrates within the disordered energy landscape of localized recombination centers. These outcomes shed light on the fundamental optical and electronic properties of functionalized germanane, enabling the groundwork for future applications in optoelectronics, light harvesting, and sensing.

Two-dimensional (2D) materials are presently one of the most actively explored platforms for the development of nanoscaled (opto)electronic devices.^1−3^ Monoelemental 2D materials (Xenes) and their substituted counterparts (Xanes, e.g., GeH or GeCH_3_) are rapidly emerging alongside the much more well-studied transition metal dichalcogenide semiconductors because of high electron mobility, a wide range of band gaps, and the possible tuning of their morphology and physical properties.^4−10^ Germanane has been proposed recently as a novel active material for optoelectronics, photoelectrocatalysis, antibacterial coating, and biosensors, with the specific performances determined by the functional groups.^11−16^

The photoluminescence (PL) of multilayer GeCH_3_, conversely to its H-terminated counterpart, is tightly linked to the presence of water in the van der Waals gap.^17^ Water intercalation switches the PL spectrum reversibly between a bright red peak centered around 1.97 eV—significantly above the 1.62 eV bandgap—for the hydrated material, and a broad band-tail emission for the dry one. The PL excitation spectrum of the 1.97 eV emission starts at 2.1 eV and has its maximum at 3.5 eV, hence demonstrating that this emission arises from strong electronic transitions involving electronic levels above the conduction band minimum and/or below the valence band maximum. The strong above-gap PL and simultaneous suppression of the band-tail emission suggest that the involved above-gap levels have no allowed relaxation channel toward the band edges. A deeper insight into the electronic nature of the involved excited states, the interplay between them, and the associated time scales^18−20^ is vital for rationalizing Xanes’ optoelectronic and light-harvesting functionalities.

In this work, we exploit time-resolved photoluminescence (TRPL) to unveil the origin of the 1.97 eV above bandgap emission in GeCH_3_. From the analysis of the emission peak energy and intensity as a function of time and temperature, we assign the observed fluorescence to the interplay of two distinct exciton populations and discuss their electronic nature.

The polycrystalline powders of GeCH_3_ were synthesized following previously established procedures.^17^

For the TRPL measurements, we used a Ti:sapphire oscillator (Chameleon Ultra II Coherent) producing a train of 140 fs pulses with a repetition rate of 80 MHz at 800 nm. A β-barium borate (BBO) crystal was used to obtain the second harmonic at 400 nm. Spatial resolution was achieved through the incorporation of a homemade microscope in the setup.^21^ A long pass dichroic mirror at 530 nm was used to reflect the excitation beam (400 nm) that was then coupled to a 20× objective (Nikon) to focus onto the sample with a spot size of about 6 μm. The emission signals were collected in backscattering geometry using a 550 nm long-pass filter and analyzed by a spectrograph (Princeton Instruments Acton SP2300) coupled to a streak camera (Hamamatsu C5680, Japan) equipped with a synchro-scan voltage sweep module. In these measurements, the fluorescence intensity was obtained as a function of both wavelength and time with spectral and temporal resolutions of ∼1 nm (∼3 meV in our spectral range) and ∼20 ps (for 2 ns time window), respectively. Cryogenic measurements were performed using a cryostat (Oxford Instruments) cooled with liquid nitrogen under vacuum conditions (10^–6^ mbar). From the synthesized powder, we selected a bulk flake of a few hundred micrometer lateral size and glued it with two thin slices of carbon tape onto a fused silica substrate. The sample was then gently annealed at 150 °C under a vacuum for 15–18 h before measurement to remove most of the intercalated water and reach a water concentration that remains stable during the measurements at varying temperatures.

Panels a and b in Figure 1 show the ball-and-stick model of dry and water intercalated bulk GeCH_3_. For the investigation of the above bandgap emission, TRPL characterization was performed as described above. Figure 1c shows the time-integrated (0–2 ns) TRPL spectra for the dry (dash-dotted blue curve) and the hydrated (solid turquoise curve) sample. The fluorescence is largely quenched but still clearly detectable when the sample is placed in 1 × 10^–6^ mbar, confirming that the remaining H_2_O molecules are enough to induce the 1.9 eV emission.^17^

**Figure 1:**
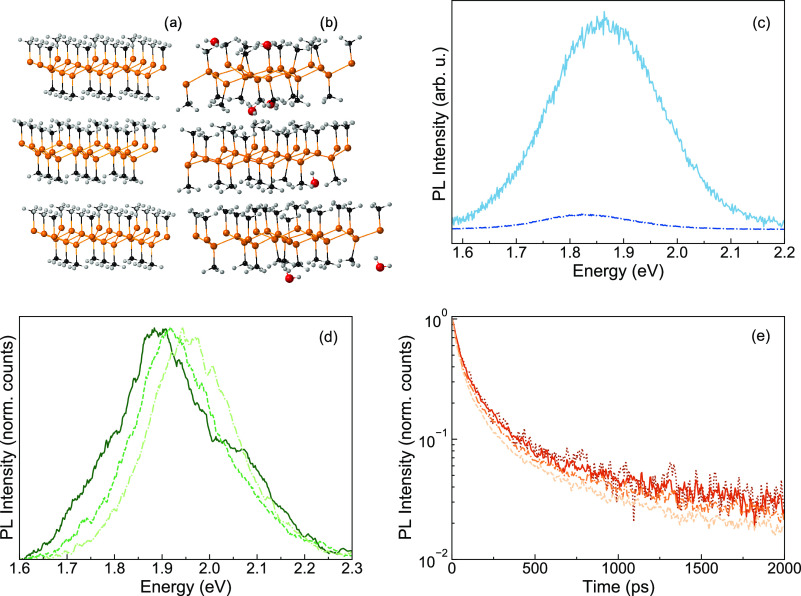
Atomic structure of GeCH_3_ (a) without water intercalation and (b) with water intercalation. Ge atoms are orange, Carbon atoms are black, hydrogen atoms are white, and oxygen atoms are red. (c) Photoluminescence integrated over time for dry (under a vacuum, dash-dotted blue curve) and hydrated (in air, turquoise solid curve) GeCH_3_ acquired with a mean excitation power of 15 μW; (d) normalized TRPL spectra of the vacuum-treated “dry” sample at 77 K integrated into the 0 < *t* < 100 ps (dash-dotted curve), 200 ps < *t* < 300 ps (dashed curve) and 900 < *t* < 1000 ps (solid curve) temporal windows; (e) normalized spectrally integrated (1.65–2.25 eV) dynamics of the vacuum-treated “dry” sample at 77 K upon mean excitation powers of 3 μW (dots), 10 μW (solid curve), 30 μW (dash-dotted curve), and 100 μW (dashed curve).

The fluorescence of the vacuum-treated “dry” sample integrated into different temporal windows (0 < *t* < 100 ps, 200 ps < *t* < 300 ps and 900 < *t* < 1000 ps), shown in Figure 1d, changes shape and peak position with time *t* after excitation, indicating that the fluorescence originates from more than just one population of recombining e–h pairs. In this respect, previous TRPL measurements on GeCH_3_ flakes revealed the presence of two emitting species, therein assigned to midgap trap states and the band tail emission.^22^ The PL traces integrated over the spectral range 1.65–2.25 eV, as shown in Figure 1e, decay almost independently of the excitation fluence over 2 orders of magnitude. We deduce that the relevant relaxation processes are linear with the density of photogenerated population *n*, i.e., follow an exponential decay and do not involve any interaction between nongeminate photoexcited species. Indeed, if free charge carriers were photogenerated, we would expect a more noticeable change in the recombination dynamics with increasing fluence, due to the rate proportional to *n*^2^ of such dynamics.^23^ Therefore, our observation is consistent with the prediction of excitons with hundreds of meV binding energy as the primary photoexcited species in germanane,^24,25^ as observed in other 2D semiconductors.^26−29^

To gain further insight into the electronic nature and the recombination dynamics of the emitting states we explored the temperature dependence of TRPL from 77 to 323 K. Figures 2a and 2b show the spectrally integrated PL traces. Remarkably, the dynamics depend nonmonotonically on temperature. In the range from 77 K to approximately 200 K, the dynamics become gradually slower with increasing temperature, while at higher temperatures they quickly become faster again.

**Figure 2:**
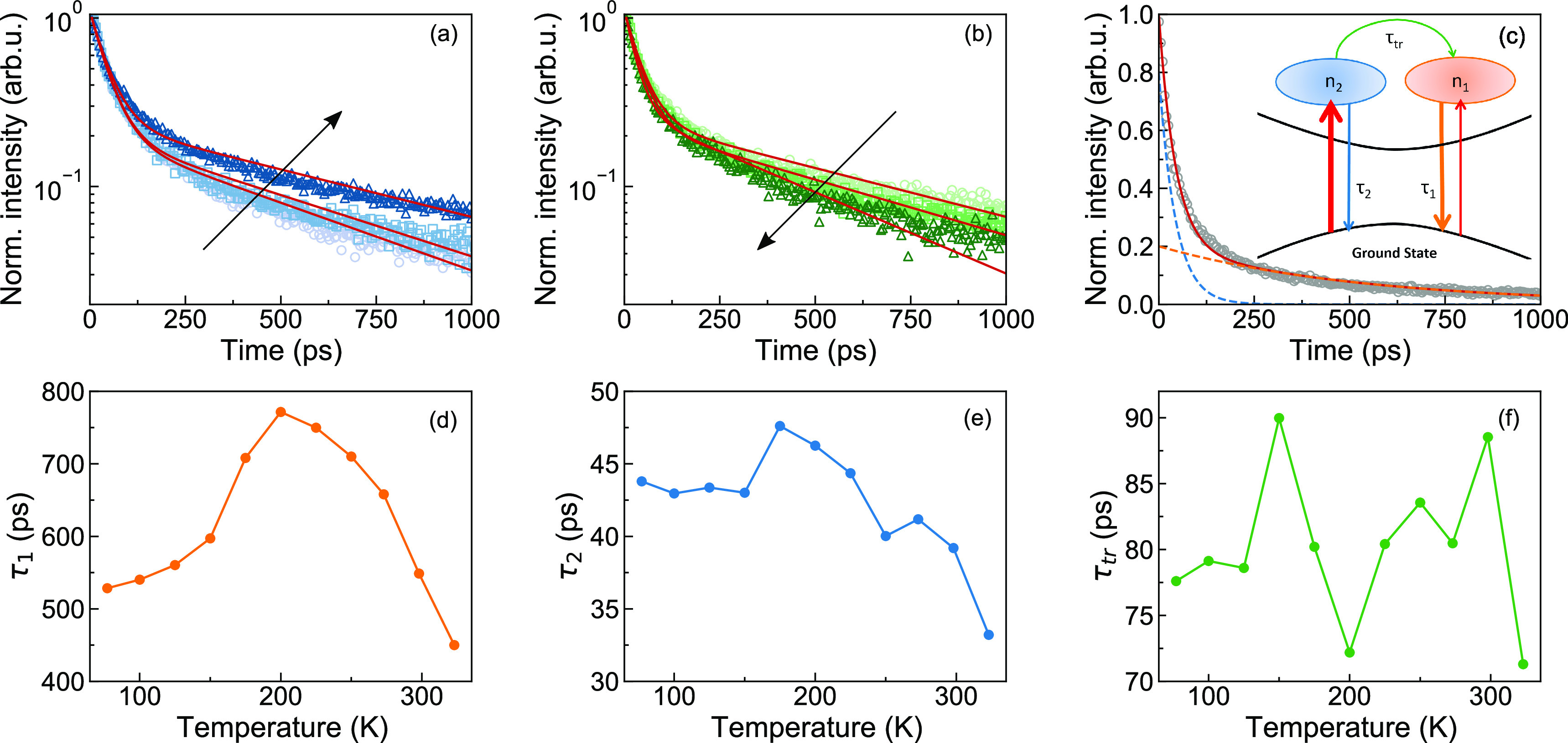
(a) Spectrally integrated dynamics acquired upon 10 μW excitation power at 77 K (circles), 150 K (squares), and 200 K (triangles) together with the fit to eq 1 (continuous red curve). (b) Spectrally integrated dynamics acquired upon 10 μW excitation power at 225 K (circles), 273 K (squares), and 323 K (triangles) together with the fit to eq 1 (continuous red curve). The black arrows indicate increasing temperature; (c) spectrally integrated dynamics acquired at 77 K (circles) upon 10 mW excitation power, fitted to eqs1 (continuous red curve), time-dependent populations of long- and short-lived excitons (orange and blue dashed lines, respectively); (d–f) time constants τ_1_, τ_2_, and τ_tr_ extracted from the fit to eq 1 as a function of temperature.

We propose a simple model for the temporal evolution of the fluorescence, sketched in the inset of Figure 2c and formulated in terms of rate equations (eq 1). We assume two distinct exciton populations *n*_1_ and *n*_2_, both localized at water-induced recombination centers (RCs). Each population is formed at a time scale shorter than our instrument response function and decays exponentially with its own time constant τ_1_ and τ_2_, which comprise both radiative and nonradiative recombination. Additionally, we assume transfer from *n*_2_ to *n*_1_ with a simple, Arrhenius-like thermal activation with a prefactor 1/τ_*tr*_ and activation energy Δ*ε*_1_:^29^
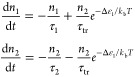
1The simple model fits the measured PL temporal evolution at different temperatures remarkably well (Figures 2a–c). For all temperatures, we obtained the best fit for initial populations of approximately 20% *n*_1_ and 80% *n*_2_. The exponential decay times are shown in Figure 2d, e, around 650 ps for τ_1_ and around 45 ps for τ_2_. These times vary by ±20% up to 300 K. Each of them comprises the radiative and nonradiative contributions τ_r_ and τ_nr_. Hence, the small variations in τ_1_ and τ_2_, which both seem to peak around 200 K, may be due to small opposite trends of τ_r_ and τ_nr_ with temperature, resulting in weakly temperature-dependent PL quantum yields η_1_ = τ_nr1_/(τ_nr1_ + τ_r1_) and η_2_ = τ_nr2_/(τ_nr2_ + τ_r2_). τ_tr_ shown in Figure 2f is a prefactor to the Arrhenius term for the thermally activated transfer of excitons from population *n*_2_ to *n*_1_. The almost constant τ_tr_ around 80 ps confirms the assumed simple Arrhenius behavior with a fitted activation energy of Δ*ε*_1_ = 52 meV. A possible back transfer of population from *n*_1_ to *n*_2_ cannot be distinguished in our data due to the short lifetime of *n*_2_.

To deconvolve the spectral contribution of *n*_1_ and *n*_2_, we fitted the time-dependent fluorescence intensity *I*(*E*, *t*) at each wavelength as
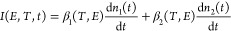
2where β_1_(*T*,*E*) = *αη*_1_(*T*)*A*_1_(*E*) and β_2_(*T*,*E*) = *αη*_2_(*T*)*A*_2_(*E*). Because all decay processes are exponential, the derivatives in eq 2 are proportional to the respective populations. Each recombining exciton emits a photon with a probability η_1_(*T*) and η_2_(*T*), respectively, corresponding to the temperature-dependent PL quantum yields of the two populations. Each emitted photon triggers a count on the detector with a probability α, which depends on the geometry of the sample and the measuring instrument and is assumed constant throughout all measurements. *A*_1_(*E*) and *A*_2_(*E*) are dimensionless functions whose integral over the whole spectral range is normalized to 1 and that reflects the shape of the PL spectra.

The fluorescence spectra of both populations η_1_(*T*)*A*_1_(*E*) and η_2_(*T*)*A*_2_(*E*), shown in Figure 3a–c are similar to single Gaussians with a width σ of around 100 meV. If we observed a simple relaxation or exciton migration within an energy distribution of recombination centers, this would always result in a red shift for increasing time *t*. In such a situation, our model would always yield *A*_1_(*E*) red-shifted relative to *A*_2_(*E*). However, *A*_1_(*E*), which is the spectrum of the longer-lived exciton species, is red-shifted relative to *A*_2_(*E*) at 100 and 323 K, whereas at 200 K it is blue-shifted. This confirms that we indeed observe two distinct populations of emitters with different temperature-dependent spectra.

**Figure 3:**
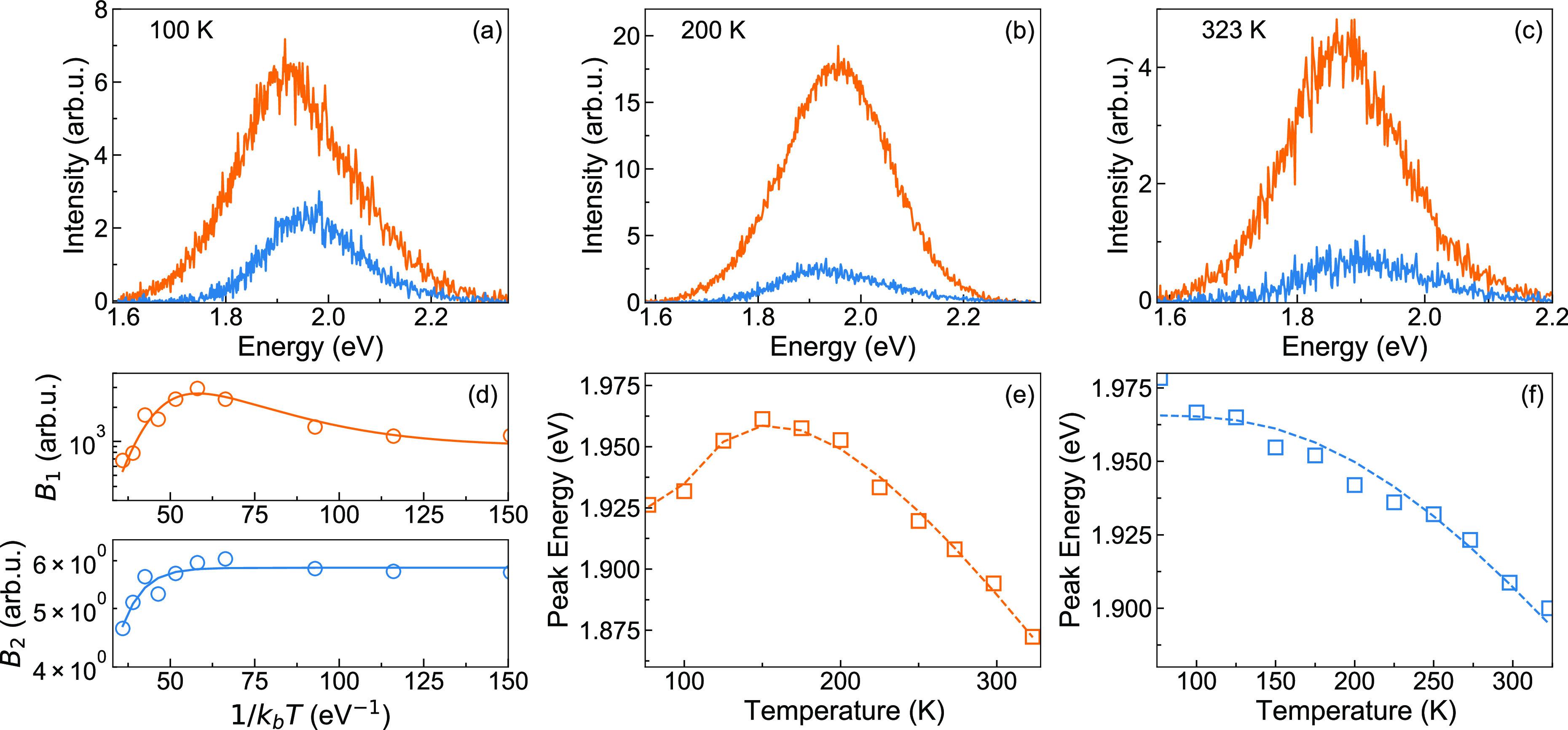
(a–c) Fluorescence spectra of long- (orange curve) and short-lived (blue curve) excitons at 100, 200, and 323 K, respectively. (d) Arrhenius plot of the integrated intensities *B*_1_ of long-lived centers (top panel, orange circles) together with the fit to eq 4 (solid orange curve) and *B*_2_ of short-lived centers (bottom panel, blue circles) together with the fit to eq 3 (solid blue curve). (e, f) Shift as a function of the temperature of long-lived centers (orange squares) together with the fit to eq 6 (dashed orange curve) and the short-lived centers (blue squares) together with the fit to eq 5 (dashed blue curve), respectively.

Given that the fluorescence spectra of the two populations as plotted in Figures 3c are proportional to the PL quantum yields, we plot *B*_1_(*T*) = ∫β_1_(*T*,*E*)d*E* and *B*_2_(*T*) = ∫β_2_(*T*,*E*)d*E* in Figure 3d to reveal additional nonradiative recombination channels. Remarkably, *B*_2_(T) suggests an Arrhenius-like thermally activated nonradiative channel:
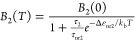
3where *B*_2_(0) is proportional to the quantum yield at 0 K, τ_nr2_ is the Arrhenius prefactor of the additional thermally activated nonradiative channel, and Δε_nr2_ is the activation energy.

Fitting our results with eq 3, we obtain τ_*nr2*_ ∼ 0.5 ps and Δε_nr2_ = 110 meV.

*B*_1_(*T*), on the other hand, starts to increase from 125 K, reaches its maximum value at 200 K, and then is quenched at higher temperatures. *n*_1_ is populated predominantly from *n*_2_ via thermal activation. Together with the introduction of an Arrhenius-type nonradiative term, this results in a more complex temperature-dependent quantum yield:
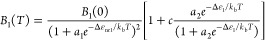
4where *B*_1_(0) is proportional to the quantum yield at 0 K, Δε_1_ and Δε_nr1_ are the activation energies of the transfer from *n*_2_ to *n*_1_ and the nonradiative processes for *n*_1_, respectively, *a*_1_*= τ*_1_*/*τ_nr1_, *a*_2_ = τ_2_*/*τ_tr_, and *c* is the ratio between the initial populations *n*_2_ and *n*_1_.

This model is adapted from the one developed for a system including two emitter populations where (i) the carrier can recombine to the ground state from each of the populations; (ii) the species can migrate only from one population to the other and not vice versa.^29^ We note that this formalism has been developed for CW PL data. Because the CW PL intensity is proportional to the PL quantum yield, we can apply the same formalism to model our *B*_1_*(T)*.

From the fit, we obtain Δε_1_ ∼ 45 meV, which is in good agreement with the value of 52 meV obtained from the fit of the spectrally integrated dynamics with eq 1. For the nonradiative processes, we extracted τ_nr1_ ∼ 2 ps and an activation energy Δε_nr1_ ∼ 140 meV. The *a*_2_ ratio between τ_2_ and τ_tr_ extracted from the fit is ∼5, which is higher than the one obtained from the fit of the dynamics to eq 1 without this additional nonradiative process (*a*_2_ ∼ 2), whereas for *c*, we obtained *n*_1_ = 12% and *n*_2_ = 88%, in good agreement with the initial populations used to solve the rate equations (20%, 80%). The agreement between the fit parameters could be improved by iterating through eqs 1–4, but no added understanding would be gained. The activation energies of nonradiative processes of both *n*_1_ and *n*_2_ are ∼3 times that of the transfer from *n*_2_ to *n*_1_. It is plausible that this activation involves either excitation into different bands within the crowded band structure of GeCH_3_,^17^ from which nonradiative recombination occurs, or thermally activated exciton dissociation. In the latter case, the activation energy would be a measure of the exciton binding energy.

For further insight into the nature of the two exciton populations, we fit *A*_1_(*E*) and *A*_2_(*E*) with a Gaussian curve and plot the peak positions as a function of the temperature in Figure 3e, f. The *A*_2_(*E*) peak position follows the O’Donnell and Chen model,^30^ which is a refinement of the empirical Varshni equation^31^ and provides more insight into the electron–phonon coupling at the origin of the temperature-dependent bandgap:
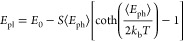
5where *E*_0_ is the PL peak at 0 K, *S* is the Huang–Rhys parameter, and ⟨*E*_ph_⟩ is the average energy of phonons coupling to the involved electronic levels. From the fit, we obtain *E*_0_ = 1.97 eV, *S* = 5.3, and ⟨*E*_ph_⟩ = 66 meV.

The temperature-dependent peak position of *A*_1_(*E*) exhibits first a blue shift from 77 to 150 K and a subsequent red shift from 175 to 323 K. Such “S shape” behavior has been previously reported for the high energy band of the GeCH_3_ PL emission,^22^ but it was not investigated in detail. The initial blue shift followed by a red shift of the emission peak energy has been observed in both CW and TRPL measurements for different semiconductor systems,^29,32−39^ including excitonic materials with a certain amount of disorder, such as organic–inorganic lead-halide perovskites,^40,41^ 2D transition metal dichalcogenides,^42−44^ or phosphorene.^45^ It has been ascribed to thermal redistribution of excitons within an ensemble of width σ of localization centers with a mean activation energy Δ*E*. We obtain the temperature-dependent PL peak position:^36^
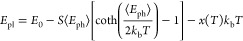
6
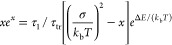
7where the first two terms of eq 6 are the same as in eq 5. The third term accounts for exciton migration between RCs,^35^ with *x*(*T*) being the solution of eq 7. τ_1_ and τ_tr_ are temperature-dependent and are taken from panels d and f in Figure 2. From the fit reported in Figure 3e, we extracted *E*_0_ = 2.1 eV, *S* = 5.2, ⟨*E*_ph_⟩= 30 meV, Δ*E* = 37 meV, and, σ = 145 meV. Hence, we can conclude that the width of the distribution is similar to the width of the PL peaks and the activation energy for exciton migration is a fraction of this width.

Our results allow us to extract vital information about the nature of the two types of excitons at the origin of the observed fluorescence. The formalism of eqs 1 and 3–7 has been developed for arrays of two species of quantum wells (QWs),^29,32−39^ where both species of QWs show a certain distribution of exciton energy. Analogously, germanane provides a disordered energy landscape for exciton migration. The temperature-dependent *A*_1_(*E*) and *A*_2_(*E*) peak positions suggest that exciton migration is relevant only for the longer-lived exciton species.

Intriguingly, the temperature dependence of *A*_1_(*E*) and *A*_2_(*E*) as described in eqs 5–7 arises from coupling to phonons with mean energy ⟨*E*_ph_⟩ = 30 meV (240 cm^–1^) for *A*_1_(*E*) and ⟨*E*_ph_⟩ = 66 meV (530 cm^–1^) for *A*_2_(*E*), suggesting that the two excitons preferentially interact with different vibrational modes of the lattice. The presence of the 1.97 eV emission after annealing suggests that the residual water bears sufficient concentration to change the electronic structure locally and to provide a high density of recombination centers that enables exciton migration between them. Quantum chemical calculations^17^ have found a dense ensemble of electronic levels close to the valence and conduction band edges as a consequence of the small local structural distortions in each layer induced by the presence of H_2_O. We can thus assume two types of emitting excitons that have their electrons and/or holes in different levels from this ensemble. Our results prescribe the following requirements for the two emitting states: (i) both have an allowed transition to the ground state but not toward the band edges, (ii) one of them can transition to the other via thermal activation, and (iii) they couple with different lattice modes. Concerning the transition from *n*_2_ to *n*_1_, the energy difference between the *A*_1_(*E*) and *A*_2_(*E*) peaks in panels e and f in Figure 3 varies strongly with temperature. This variation is inconsistent with a simple Arrhenius-like thermally stimulated transfer with a fixed activation energy of 37 meV, as assumed in eq 1 and confirmed in Figure 3f. An alternative mechanism has recently been proposed for the interlayer exciton recombination in a heterostructure of two 2D monolayers.^46^ Low energy phonons periodically modulate the band structure between a direct and an indirect gap, leading to a recombination rate that has a temperature dependence very similar to an Arrhenius behavior with formal activation energy much higher than the energies of the phonons involved. We can assume a similar mechanism for the population transfer from *n*_2_ to *n*_1_ via a phonon-induced modulation of the band structure.

To summarize, we used TRPL spectroscopy at different temperatures to study the above bandgap fluorescence of GeCH_3_ samples. We find two distinct populations of emitting excitons localized at RCs within the intercalated water. Compared to 2D transition metal dichalcogenides, research on Xenes and Xanes is still in its infancy and the exciton binding energy, exciton transport mechanisms, trions, biexcitons, and higher many-body effects still need investigation, as well as fluorescence quantum yield and charge separation at interfaces as the groundwork for future applications in optoelectronics, light harvesting, and sensing.
